# Calixapap: Calixarene-based Cluster of Acetaminophen as a Novel Antiradical Agent

**Published:** 2019

**Authors:** Amirali Delnavaz Shahr, Fazel Nasuhi Pur, Karim Akbari Dilmaghani

**Affiliations:** a *Department of Chemistry, Faculty of Science, Urmia University, Urmia, Iran. *; b *bHealth Technology Incubator Center, Urmia University of Medical Sciences, Urmia, Iran*

**Keywords:** Acetaminophen, Paracetamol, Calixarene, Cluster, Antiradical, Radical scavenger

## Abstract

In this paper, the synthesis and free-radical scavenging capacity of novel calix[4]arene-based cluster of paracetamol was reported. The phenolic structures of acetaminophen and calix[4]arene prompted us for designing a synthetic route for calix[4]arene-based cyclic tetramer of paracetamol. The present chalice-shaped cluster is the first example of calixarene/acetaminophen hybrid and paracetamol can be considered as ¼ of the synthetic cyclic tetramer. Free-radical scavenging tests were determined by the 2,2-diphenyl-1-picrylhydrazyl radical in methanol. The results of antiradical-testing showed the enhanced free-radical scavenging capacity (~ 10-fold) for the prepared chaliced-shaped cluster with respect to the corresponding single therapeutic drug unit (acetaminophen). It is maybe attributed to the multivalency, spatial preorganization, and synergistic effect of four impacted drug units in the cluster structure (clustering effect).

## Introduction

Acetaminophen as known as paracetamol or acetyl-para-aminophenol (APAP) is one of the most popular and world-spread antipyretic and analgesic drug (pain-killer) with some other medical applications such as anti-inflammatory, antirheumatic, anticancer, and antioxidant activities (1-3). Due to the phenolic structure (free hydroxyl group) of APAP, it is a potential antiradical for scavenging free-radicals such as 2,2-diphenyl-1-picrylhydrazyl radical (DPPH•) (3).

On the other hand, calixarenes are suitable structures for designing and developing of new drugs via clustering of the single drug units on them (4-9). So, in our continuous studies to the synthesis of new calixdrugs (9), we wish to report a novel calix[4]arene-based cluster for the presentation of APAP cyclic tetramer and its antiradical activity in comparison to APAP alone as reference drug. Based on calixarenes’ nomenclature (9), the chaliced shape of APAP was innovatively named calixapap. 

## Experimental

*General*

The melting points of all compounds were determined on a Philip Harris C4954718 apparatus without calibration. The IR spectra were measured in KBr disks on a Thermo Nicolet 610 Nexus FT-IR spectrometer. The ^1^H NMR (300 MHz) and ^13^C NMR (75 MHz) measurements were performed on a Bruker AM-300 spectrometer in DMSO-d_6_ using TMS as the internal reference. Elemental analyses were carried out on a PerkinElmer 240c analyzer. Mass spectra were recorded on a JEOL-JMS 600 (FAB MS) instrument. All chemicals were purchased from Merck and Aldrich (Tehran, Iran) and used as received.

*Chemistry*

Compound **1** was prepared by the previously reported method as white powder (10). 

*Synthesis of 1,3-alternate5,11,17,23-Tetraacetyl-calix[4]arene (2)*

A mixture of compound **1** in 1,3-alternate conformer (1 g), AlCl_3_ (1.35 g) in PhNO_2_ (30 mL) was stirred under N_2_ atmosphere at room temperature for 2 h and then stirred for 6 h at 65 ˚C. The reaction mixture was cooled to 0 ˚C and mixed with HCl 10% (15 mL) at 0 ˚C. The organic layer was separated, washed with water and then dried over MgSO_4_. After evaporation of the organic solvent, the residue was refluxed in acetone (10 mL) for 0.5 h followed by cooling to room temperature for sedimentation of white solid. Crystallization from CHCl_3_ afforded compound **2** as white crystals.

Compound **2**: yield, 70%; m.p. > 300 °C; ^1^H NMR (300 MHz; DMSO-d_6_; δ, ppm): 9.42 (s, 4H, ArOH), 6.72-6.75 (m, 8H, ArH), 3.97 (s, 8H, ArCH_2_Ar), 2.51 (s, 12H, CH_3_); ^13^C NMR (75 MHz; DMSO-d_6_; δ, ppm): 195.95 (C=O), 156.7 (C*i*), 129.5 (C*m*), 129.4 (C*o*), 129.3 (Cp), 37.1 (ArCH_2_Ar). 26.4 (CH_3_); Anal. Calcd. for C_36_H_32_O_8_: C, 72.96; H, 5.44; O, 21.60 Found: C, 73.01; H, 5.43; O, 21.56; FAB-MS (m/z): 592.27 (M+).

*Synthesis of 1,3-alternate Calixapap (3)*

A mixture of compound **2** (0.5 g) and fuming sulfuric acid (1.5 mL) was stirred under N_2_ atmosphere at room temperature and then sodium azide (0.27 g) was added slowly over 0.5 h at the same conditions. The mixture was stirred for 6 h at 60 ˚C under N_2_ atmosphere. The reaction mixture was then cooled to 0 ˚C and stirred (0.5 h) for sedimentation. The residue was filtered and washed with water to give white solid. Crystallization from DMF gave compound **3** as white crystals.

Compound **3**: yield, 72%; m.p. > 300 °C; ^1^H NMR (300 MHz; DMSO-d_6_; δ, ppm): 9.54 (s, 4H, NH), 9.31 (s, 4H, ArOH), 7.19-7.22 (m, 8H, ArH), 3.77 (s, 8H, ArCH_2_Ar), 1.93 (s, 12H, CH_3_); ^13^C NMR (75 MHz; DMSO-d_6_; δ, ppm): 167.7 (C=O), 145.3 (C*i*), 133.2 (C*m*), 127.4 (C*o*), 119.1 (Cp), 36.9 (ArCH_2_Ar). 23.8 (CH_3_); Anal. Calcd. for C_36_H_36_N_4_O_8_: C, 66.25; H, 5.56; N, 8.58; O, 19.61 Found: C, 66.23; H, 5.55; N, 8.60; O, 19.62; FAB-MS (m/z): 652.29 (M+).

## Results and Discussion

**Scheme 1:**
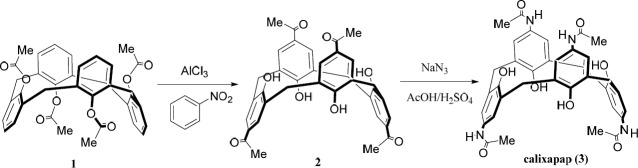
Synthetic route of calixapap

The synthetic pathway to calixapap is depicted in Scheme 1. The synthetic route included the conversion of compound **1** to **2** by the Fries rearrangement in the presence of nitrobenzene as solvent and AlCl_3_ as catalyst. Calixapap as the final product was obtained by the insertion of four nitrogen atoms to compound **2** in the presence of oleum/AcOH via using NaN_3_. Because of the 1, 3-alternate conformation of compound **1**, compounds **2,** and **3** (as its derivatives) are in 1,3-alternate conformation, too. It can be confirmed by chemical shifts (~ 37 ppm) of the carbons of the methylene bridges in their ^13^C NMR spectra and the singlet pattern for protons of the methylene bridges in their ^1^H NMR spectra.

**Table 1:** Stoichiometry factor n for reactions of hydrogen atom transfer from antiradical to DPPH (DPPH/Antiradical Molar Ratio = 4, MeOH, 25 °C).

Compound	Factor n at 600 (sec.)
**APAP**	0.55
**Calixapap**	5.28
**Calixtyrosol**	4.83 (ref. 7)
**DHPM cluster**	2.71 (ref. 8)

**Figure 1:**
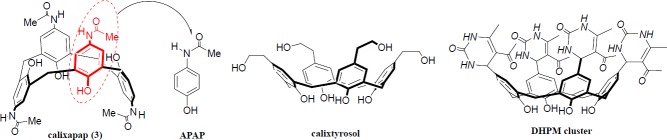
Structural comparison of APAP and calixapap and two other synthetic calixarene-based antiradicals

In order to evaluate the potentially amplified antiradical activity of calixapap, it was compared with its monomer, paracetamol, as reference compound. The *in-vitro* free-radical scavenging activities of compounds were determined for 2,2-diphenyl-1-picrylhydrazyl radical (DPPH•) using our previously published procedure (7, 8). The results of these tests are shown in Table 1 based on factor n as the number of DPPH• radicals quenched per antiradical molecules. The solutions of antiradicals were prepared in methanol. The stoichiometric factor n was calculated (for DPPH/Antiradical molar ratio = 4) by the following formula: n = (∆A_515_/ε_515_)/C 

where ∆A_515_ = A_0_ – A_f_ is the absorbance difference between the initial and stationary state of DPPH• solution, and ε_515_ = 11240 M^–1^ cm^–1^ (molar absorption coefficient at 515 nm assuming a purity of 95%), and C is the concentration of antiradical in cuvette at the initial moment of time. In fact, the stoichiometric factor n is the number of DPPH• radicals quenched per antiradical molecule and is characteristic of the radical inhibiting ability of scavenger. It is the best parameter for demonstration of the clustering effect on free-radical scavenging activity of the test compounds (11).

As shown in the Table 1, the stoichiometry factor n (the number of free-radicals quenched per antiradical molecule) for calixapap is more than the rest of n values for APAP, calixtyrosol and DHPM cluster (Figure 1). It means that, calixapap is more potent than the mentioned antiradicals for scavenging free-radicals.

As shown in Figure 1, paracetamol or APAP can be considered as ¼ of the chalice-shaped cyclic tetramer (calixapap).

Compared to acetaminophen, the enhanced antiradical activity (~ 10-fold) of calixapap can be explained by clustering effect, probably associated with the multivalency, spatial preorganization, and the synergistic effect of four embedded paracetamol units. In addition, the presence of methylene bridges (*ortho*-position) as bulky groups (Figure 1) maybe increase the electron density on the hydroxyl group as well as its hydrogen bonding at the lower rim of calixapap which led to stabilization of the phenoxy-radical in the structure of antiradical. All these have positive effects to reduce the rate of possible propagation reactions (7).

In summary, the present work describes the first example of calixarene-based paracetamol cluster possessing efficient and amplified free-radical scavenging activity (~ 10-fold) due to its synergistic effect of cluster arms. In fact, the most popular pain-killer drug, acetaminophen, can be considered as ¼ of chalice-shaped cyclic tetramer (calixapap).
